# Supplementary material for: Beneficial Effect of Dietary Approaches to Stop Hypertension Diet Combined with Regular Physical Activity on Fat Mass and Anthropometric and Metabolic Parameters in People with Overweight and Obesity
Source: Nutrients. 2024 Sep 20;16(18):3187. doi: 10.3390/nu16183187 (PMC11435128; doi:10.3390/nu16183187)
Supplement: Supplementary file 1 [file nutrients-16-03187-s001.zip › nutrients-3133661-supplementary.pdf]

Table S1. Anthropometric and metabolic parameters and indices at baseline and at the end of the study according to age. Significant differences are marked in ***italic bold***.

Parameter	Age <48 years		Age ≥48 years		P value between groups	P value for heterogeneity
	**Mean ± SD or n (%)**	**P value within group**	**Mean ± SD or n (%)**	**P value within group**		
Age (years)	38.9 ± 6.36		58.8 ± 7.34		***<0.001***	
Male sex (n)	9 (18.4%)		6 (11.8%)		0.519	
Hypertension (n)	19 (38.0%)		31 (62.0%)		***0.028***	
Dysglycemia (n)	12 (24.5%)		17 (33.3%)		0.451	
Standing height (cm)	167 ± 6		164 ± 9		***0.030***	
Weight (kg) baseline	93.8 ± 18.2	***<0.001***	90.6 ± 15.3	***<0.001***	0.549	0.296
Weight (kg) end of study	88.3 ± 17.0		84.7 ± 14.5		0.356	
BMI (kg/m^2) baseline	33.57 ± 4.84	***<0.001***	33.85 ± 5.02	***<0.001***	0.767	0.285
BMI (kg/m^2) end of study	31.63 ± 4.47		31.67 ± 4.84		0.983	
Waist (cm) baseline	101.5 ± 13.6	***<0.001***	103.7 ± 14.1	***<0.001***	0.436	0.531
Waist (cm) end of study	95.6 ± 13.5		98.3 ± 13.8		0.324	
WHtR baseline	0.61 ± 0.07	***<0.001***	0.64 ± 0.09	***<0.001***	0.104	0.539
WHtR end of study	0.57 ± 0.07		0.60 ± 0.09		0.119	
Hip (cm) baseline	115.9 ± 9.3	***<0.001***	115.1 ± 8.9	***<0.001***	0.748	0.668
Hip (cm) end of study	110.4 ± 8.4		109.7 ± 9.5		0.707	
WHR baseline	0.88 ± 0.09	***0.031***[†]	0.90 ± 0.10	0.259	0.104	0.823
WHR end of study	0.87 ± 0.09		0.90 ± 0.10		0.107	
Fat mass (%) baseline	38.3 ± 6.0	***<0.001***	41.8 ± 7.3	***<0.001***	***0.009***	0.319
Fat mass (%) end of study	35.0 ± 6.1		38.2 ± 7.6		***0.022***	
TC (mmol/L) baseline	5.32 ± 1.06	***<0.001***	5.43 ± 1.06	***<0.001***	0.584	0.085
TC (mmol/L) end of study	4.96 ± 0.94		4.81 ± 0.84		0.488	
TG (mmol/L) baseline	1.49 ± 0.62	***0.002***[†]	1.35 ± 0.54	***0.013***[†]	0.290	0.396
TG (mmol/L) end of study	1.29 ± 0.60		1.20 ± 0.42		0.634	
HDL-C (mmol/L) baseline	1.42 ± 0.36	0.056	1.56 ± 0.43	***0.011***[†]	0.073	0.560
HDL-C (mmol/L) end of study	1.37 ± 0.34		1.46 ± 0.34		0.150	
LDL-C (mmol/L) baseline	3.27 ± 1.01	***0.005***	3.26 ± 0.95	***<0.001***	0.789	0.085
LDL-C (mmol/L) end of study	3.00 ± 0.90		2.81 ± 0.76		0.324	
Glucose (mmol/L) baseline	5.23 ± 0.76	0.795	5.51 ± 1.09	0.188	0.055	0.268
Glucose (mmol/L) end of study	5.27 ± 0.68		5.38 ± 0.83		0.326	
Uric acid (μmol/L) baseline	308.7 ± 82.1	***0.009***[†]	323.6 ± 73.8	***0.021***[†]	0.444	0.945
Uric acid (μmol/L) end of study	293.3 ± 70.8		306.9 ± 75.5		0.355	
TG/HDL-C ratio baseline	2.68 ± 1.53	***0.032***[†]	2.29 ± 1.64	0.380	0.153	0.361
TG/HDL-C ratio end of study	2.36 ± 1.38		2.06 ± 1.13		0.404	
TyG index baseline	4.67 ± 0.22	***0.004***	4.65 ± 0.22	***0.008***[†]	0.670	0.718
TyG index end of study	4.59 ± 0.25		4.59 ± 0.18		0.923	
VAI baseline	4.61 ± 2.48	***0.012***[†]	4.09 ± 2.55	0.311	0.247	0.306
VAI end of study	3.96 ± 2.02		3.68 ± 1.73		0.596	

SD: Standard Deviation; BMI: Body mass index; WHtR: waist-to-height ratio; WHR: waist/hip ratio; TG/HDL-C: triglycerides/HDL cholesterol ratio; TyG: triglycerides/glucose index; VAI: visceral adiposity index. [†]statistical power <0.8

Table S2. Anthropometric and metabolic parameters and indices at baseline and at the end of the study according to sex. Significant differences are marked in ***italic bold***.

Parameter	Women		Men		P value between groups	P value for heterogeneity
	Mean ± SD or n (%)	P value within group	Mean ± SD or n (%)	P value within group		
Age (years)	49.46 ± 11.66		45.27 ± 14.26		0.218	
Hypertension (n)	41 (48.2%)		9 (60.0%)		0.575	
Dysglycemia (n)	21 (24.7%)		8 (53.3%)		***0.033***	
Standing height (cm)	163 ± 6		177 ± 8		***<0.001***	
Weight (kg) baseline	87.85 ± 12.81	***<0.001***	116.65 ± 16.22	***<0.001***	***<0.001***	0.222
Weight (kg) end of study	82.42 ± 11.81		109.77 ± 15.65		***<0.001***	
BMI (kg/m^2) baseline	33.06 ± 4.73	***<0.001***	37.41 ± 4.34	***<0.001***	***0.001***	0.650
BMI (kg/m^2) end of study	31.02 ± 4.41		35.21 ± 4.37		***0.001***	
Waist (cm) baseline	99.70 ± 12.12	***<0.001***	118.90 ± 11.37	***<0.001***	***<0.001***	0.477
Waist (cm) end of study	93.97 ± 11.62		114.03 ± 11.63		***<0.001***	
WHtR baseline	0.61 ± 0.08	***<0.001***	0.68 ± 0.07	***<0.001***	***0.003***	0.239
WHtR end of study	0.58 ± 0.08		0.65 ± 0.07		***0.002***	
Hip (cm) baseline	115.10 ± 8.74	***<0.001***	117.50 ± 10.75	***<0.001***	0.543	0.220
Hip (cm) end of study	109.57 ± 8.85		112.77 ± 9.14		0.220	
WHR baseline	0.865 ± 0.073	***0.027***	1.016 ± 0.105	0.794	***<0.001***	0.347
WHR end of study	0.857 ± 0.080		1.014 ± 0.103		***<0.001***	
Fat mass (%) baseline	40.85 ± 6.27	***<0.001***	35.46 ± 8.64	***<0.001***	***0.009***	0.896
Fat mass (%) end of study	37.41 ± 6.59		31.75 ± 7.95		***0.004***	
TC (mmol/L) baseline	209.97 ± 41.20	***<0.001***	193.33 ± 36.36	***0.024***†	0.259	0.692
TC (mmol/L) end of study	190.37 ± 34.99		178.73 ± 29.53		0.327	
TG (mmol/L) baseline	118.34 ± 47.52	***<0.001***	166.47 ± 55.46	0.222	***<0.001***	0.791
TG (mmol/L) end of study	103.07 ± 38.24		149.00 ± 63.73		***0.016***	
HDL-C (mmol/L) baseline	60.42 ± 14.62	***0.002***	40.60 ± 7.48	0.389	***<0.001***	0.348
HDL-C (mmol/L) end of study	57.07 ± 12.40		39.93 ± 6.36		***<0.001***	
LDL-C (mmol/L) baseline	127.27 ± 38.69	***<0.001***	119.40 ± 32.27	***0.024***†	0.626	0.496
LDL-C (mmol/L) end of study	112.53 ± 33.20		109.87 ± 26.92		0.873	
Glucose (mmol/L) baseline	95.31 ± 13.31	0.248	104.47 ± 30.16	0.552	0.240	0.225
Glucose (mmol/L) end of study	93.99 ± 10.82		106.40 ± 21.60		***0.003***	
Uric acid (μmol/L) baseline	5.05 ± 1.10	***0.007***	6.78 ± 1.48	***0.028***†	***<0.001***	0.313
Uric acid (μmol/L) end of study	4.80 ± 1.04		6.44 ± 1.33		***<0.001***	
TG/HDL-C ratio baseline	2.15 ± 1.24	***0.036***†	4.35 ± 2.07	0.263	***<0.001***	0.892
TG/HDL-C ratio end of study	1.92 ± 0.89		3.84 ± 1.80		***<0.001***	
TyG index baseline	4.63 ± 0.20	***<0.001***	4.84 ± 0.22	0.267	***<0.001***	0.838
TyG index end of study	4.56 ± 0.19		4.78 ± 0.28		***0.011***	
VAI baseline	4.06 ± 2.32	***0.013***†	6.01 ± 3.02	0.421	***0.005***	0.946
VAI end of study	3.56 ± 1.64		5.27 ± 2.46		***0.013***	

SD: Standard Deviation; BMI: Body mass index; WHtR: waist-to-height ratio; WHR: waist/hip ratio; TC – total cholesterol; TG – triglycerides; HDL-C – HDL cholesterol; LDL-C – LDL cholesterol; TyG: triglycerides/glucose index; VAI: visceral adiposity index. †statistical power <0.8

Table S3. Anthropometric and metabolic parameters and indices at baseline and at the end of the study according to presence of hypertension at baseline. Significant differences are marked in ***italic bold***.

Parameter	Hypertension		No hypertension		P value between groups	P value for heterogeneity
	Mean ± SD or n (%)	P value within group	Mean ± SD or n (%)	P value within group		
Age (years)	52.60 ± 11.07		45.06 ± 12.01		***0.002***	
Male sex (n)	9 (18.0%)		6 (12.0%)		0.575	
Dysglycemia (n)	18 (36.0%)		11 (22.0%)		0.186	
Standing height (cm)	165 ± 9		165 ± 6		0.695	
Weight (kg) baseline	96.77 ± 18.48	***<0.001***	87.57 ± 13.70	***<0.001***	***0.011***	0.148
Weight (kg) end of study	90.40 ± 17.74		82.65 ± 12.58		***0.030***	
BMI (kg/m^2) baseline	35.25 ± 5.25	***<0.001***	32.17 ± 4.04	***<0.001***	***0.001***	0.165
BMI (kg/m^2) end of study	32.94 ± 5.19		30.36 ± 3.60		***0.015***	
Waist (cm) baseline	107.43 ± 14.14	***<0.001***	97.73 ± 11.69	***<0.001***	***<0.001***	0.547
Waist (cm) end of study	102.02 ± 14.22		91.94 ± 10.99		***<0.001***	
WHtR baseline	0.65 ± 0.08	***<0.001***	0.59 ± 0.07	***<0.001***	***<0.001***	0.548
WHtR end of study	0.62 ± 0.09		0.56 ± 0.07		***<0.001***	
Hip (cm) baseline	117.12 ± 9.74	***<0.001***	113.80 ± 8.07	***<0.001***	0.189	0.981
Hip (cm) end of study	111.60 ± 9.72		108.50 ± 7.83		0.205	
WHR baseline	0.92 ± 0.10	0.406	0.86 ± 0.08	***0.027***[†]	***0.002***	0.366
WHR end of study	0.91 ± 0.10		0.85 ± 0.09		***<0.001***	
Fat mass (%) baseline	41.20 ± 7.41	***<0.001***	38.88 ± 6.22	***<0.001***	0.094	0.788
Fat mass (%) end of study	37.65 ± 7.62		35.48 ± 6.36		0.126	
TC (mmol/L) baseline	5.24 ± 1.06	***<0.001***	5.50 ± 1.05	***<0.001***	0.214	0.340
TC (mmol/L) end of study	4.79 ± 0.89		4.98 ± 0.89		0.389	
TG (mmol/L) baseline	1.51 ± 0.64	***0.003***	1.32 ± 0.50	***0.006***[†]	0.111	0.627
TG (mmol/L) end of study	1.29 ± 0.48		1.20 ± 0.55		0.200	
HDL-C (mmol/L) baseline	1.46 ± 0.44	***0.042***[†]	1.51 ± 0.36	***0.010***[†]	0.364	0.641
HDL-C (mmol/L) end of study	1.41 ± 0.37		1.42 ± 0.32		0.664	
LDL-C (mmol/L) baseline	3.13 ± 0.94	***0.007***	3.40 ± 1.01	***<0.001***	0.244	0.301
LDL-C (mmol/L) end of study	2.81 ± 0.78		3.00 ± 0.88		0.276	
Glucose (mmol/L) baseline	5.63 ± 1.19	0.124	5.11 ± 0.50	0.495	***0.023***	0.228
Glucose (mmol/L) end of study	5.49 ± 0.94		5.16 ± 0.47		0.109	
Uric acid (μmol/L) baseline	325.4 ± 81.5	***0.039***[†]	306.3 ± 74.4	***0.009***	0.426	0.686
Uric acid (μmol/L) end of study	311.7 ± 79.1		287.9 ± 66.0		0.186	
TG/HDL-C ratio baseline	2.75 ± 1.82	***0.041***[†]	2.22 ± 1.29	0.310	0.099	0.293
TG/HDL-C ratio end of study	2.32 ± 1.31		2.10 ± 1.22		0.347	
TyG index baseline	4.71 ± 0.23	***<0.001***	4.61 ± 0.19	***0.035***[†]	***0.020***	0.350
TyG index end of study	4.63 ± 0.22		4.55 ± 0.21		0.085	
VAI baseline	5.42 ± 3.81	***0.031***	4.21 ± 2.55	0.150	0.057	0.422
VAI end of study	4.56 ± 2.80		3.89 ± 2.37		0.258	

SD: Standard Deviation; BMI: Body mass index; WHtR: waist-to-height ratio; WHR: waist/hip ratio; TC – total cholesterol; TG – triglycerides; HDL-C – HDL cholesterol; LDL-C – LDL cholesterol; TyG: triglycerides/glucose index; VAI: visceral adiposity index. [†]statistical power <0.8

Table S4. Anthropometric and metabolic parameters and indices at baseline and at the end of the study according to presence of dysglycemia at baseline. Significant differences are marked in ***italic bold***.

Parameter	Dysglycemia		Normoglycemia		P value between groups	P value for heterogeneity
	Mean ± SD or n (%)	P value within group	Mean ± SD or n (%)	P value within group		
**Age (years)**	**52.48 ± 12.63**		47.34 ± 11.63		0.053	
Male sex (n)	8 (27.6%)		7 (9.9%)		***0.033***	
Hypertension (n)	18 (62.1%)		32 (45.1%)		0.186	
Standing height (cm)	165 ± 10		165 ± 7		0.772	
Weight (kg) baseline	99.6 ± 18.5	***<0.001***	89.1 ± 15.2	***<0.001***	***0.004***	0.397
Weight (kg) end of study	93.4 ± 18.3		83.7 ± 13.8		***0.011***	
BMI (kg/m^2) baseline	36.24 ± 5.25	***<0.001***	32.67 ± 4.39	***<0.001***	***0.002***	0.487
BMI (kg/m^2) end of study	33.98 ± 5.16		30.70 ± 4.06		***0.002***	
Waist (cm) baseline	110.2 ± 14.9	***<0.001***	99.5 ± 12.1	***<0.001***	***<0.001***	0.573
Waist (cm) end of study	104.3 ± 14.9		94.0 ± 11.9		***<0.001***	
WHtR baseline	0.67 ± 0.08	***<0.001***	0.60 ± 0.07	***<0.001***	***<0.001***	0.576
WHtR end of study	0.63 ± 0.08		0.57 ± 0.07		***<0.001***	
Hip (cm) baseline	117.0 ± 9.9	***<0.001***	114.8 ± 8.7	***<0.001***	0.378	0.661
Hip (cm) end of study	111.7 ± 10.6		109.4 ± 8.1		0.424	
WHR baseline	0.94 ± 0.10	0.368	0.87 ± 0.09	0.070	***<0.001***	0.894
WHR end of study	0.93 ± 0.10		0.86 ± 0.09		***<0.001***	
Fat mass (%) baseline	43.3 ± 7.0	***<0.001***	38.7 ± 6.5	***<0.001***	***0.002***	0.434
Fat mass (%) end of study	39.6 ± 7.5		35.3 ± 6.6		***0.005***	
TC (mmol/L) baseline	5.46 ± 1.37	***0.002***	5.34 ± 0.91	***<0.001***	0.787	0.927
TC (mmol/L) end of study	4.95 ± 1.17		4.86 ± 0.75		0.817	
TG (mmol/L) baseline	1.59 ± 0.68	***0.007†***	1.35 ± 0.52	***0.012†***	0.090	0.445
TG (mmol/L) end of study	1.35 ± 0.51		1.20 ± 0.52		0.133	
HDL-C (mmol/L) baseline	1.41 ± 0.42	0.092	1.52 ± 0.39	***0.004***	0.115	0.915
HDL-C (mmol/L) end of study	1.32 ± 0.32		1.45 ± 0.34		***0.042***	
LDL-C (mmol/L) baseline	3.32 ± 1.22	***0.015†***	3.24 ± 0.87	***<0.001***	0.976	0.582
LDL-C (mmol/L) end of study	3.02 ± 1.13		2.86 ± 0.68		0.770	
Glucose (mmol/L) baseline	6.25 ± 1.24	0.099	5.01 ± 0.44	0.768	***<0.001***	0.087
Glucose (mmol/L) end of study	6.02 ± 0.98		5.03 ± 0.37		***<0.001***	
Uric acid (μmol/L) baseline	350.0 ± 90.1	***0.045†***	302.1 ± 68.5	***0.005***	***0.005***	0.852
Uric acid (μmol/L) end of study	332.9 ± 84.8		286.5 ± 63.9		***0.004***	
TG/HDL-C ratio baseline	2.96 ± 1.97	0.141	2.29 ± 1.37	0.120	0.070	0.471
TG/HDL-C ratio end of study	2.56 ± 1.31		2.07 ± 1.23		***0.039***	
TyG index baseline	4.78 ± 0.24	***0.008†***	4.61 ± 0.19	***0.003***	***<0.001***	0.406
TyG index end of study	4.70 ± 0.23		4.55 ± 0.20		***0.003***	
VAI baseline	5.06 ± 3.14	0.100	4.06 ± 2.17	0.051	0.152	0.541
VAI end of study	4.34 ± 1.95		3.61 ± 1.82		0.053	

SD: Standard Deviation; BMI: Body mass index; WHtR: waist-to-height ratio; WHR: waist/hip ratio; TC – total cholesterol; TG – triglycerides; HDL-C – HDL cholesterol; LDL-C – LDL cholesterol; TyG: triglycerides/glucose index; VAI: visceral adiposity index. †statistical power <0.8
